# Low-Cost Laser-Acoustic PVC Identification System Based on a Simple Neural Network

**DOI:** 10.3390/s22208035

**Published:** 2022-10-21

**Authors:** Eric Timmermann, Philip Geißler, Robert Bansemer

**Affiliations:** Leibniz Institute for Plasma Science and Technology (INP Greifswald), Felix-Hausdorff-Straße 2, 17489 Greifswald, Germany

**Keywords:** laser acoustic, laser cutting, neural network, polymer classification, plastics, safety, sorting

## Abstract

Desktop laser cutters are an affordable and flexible rapid-prototyping tool, but some materials cannot be safely processed. Among them is polyvinyl chloride (PVC), which users usually cannot distinguish from other, unproblematic plastics. Therefore, an identification system for PVC applicable in a low-cost laser cutter has been developed. For the first time, this approach makes use of the laser-ablative sound generated by a low-power laser diode. Using a capacitor microphone, a preprocessing algorithm and a very simple neural network, black PVC could be detected with absolute reliability under ideal conditions. With ambient noise, the accuracy dropped to 80%. A different color of the material did not influence the accuracy to detect PVC, but a susceptibility of the method against a color change was found for other materials. The ablation characteristics for different materials were recorded using a fast-framing camera to get a better insight into the mechanisms behind the investigated process. Although there is still potential for improvements, the presented method was found to be promising to enhance the safety of future desktop laser cutters.

## 1. Introduction

In recent years, laser cutting has developed from an industry-only technology to very cost-effective “desktop laser cutters”, which are affordable to many nonprofessionals. However, the handling of these devices also involves significant dangers for the untrained user. In addition to radiation, the risk from harmful or corrosive exhaust gases is often disregarded. While particulate and active carbon filters protect users from the hazardous exhaust fumes, practical experience has revealed another problem: halogenated plastics, especially the widespread polyvinyl chloride (PVC), release large quantities of hydrogen chloride during processing, which is highly corrosive and destroys the laser cutter from the inside in a very short time. The situation is aggravated by the fact that most users cannot distinguish PVC from other (unproblematic) plastics such as polyethylene (PE). 

This problem motivates the search for an automated PVC identification method. To do this in an application-oriented manner, a commercial desktop laser cutter (“dreamcut 2”, Mr Beam Lasers GmbH, Munich, Germany) with a blue (448 nm) laser diode with an optical output power of 3.5 W is used as a device under test. The concept intended in this contribution checks whether PVC is inserted at the beginning of each laser-cutting process. If this is the case, the cutting process does not start at all. As a typical cutting process lasts for minutes or hours, the PVC identification system does not need to be fast or real-time capable. On the other hand, the intended system needs to be very cost-effective. 

Existing solutions do not meet these requirements because they were created for other use cases. Established technologies for plastic sorting, which essentially exploit their (different) optical properties in the near-infrared range [1], mostly fail with (black-)colored plastics [2]. Further developments, such as procedures based on mid-infrared spectroscopy [3], require significantly more expensive hardware. 

Furthermore, laser-induced breakdown spectroscopy (LIBS) was proven for its polymer classification capabilities [4]. Laser-based methods are in principle interesting here, as the laser cutter already has an existing laser source. However, the power of the laser diode is not sufficient to put the material into the plasma state needed for LIBS. Nevertheless, it may be powerful enough to excite the material to thermomechanical vibrations [5]—A phenomenon known as laser-, or more generally, photo- or opto-acoustics.

However, only a tiny fraction of the photoacoustic discipline is relevant here. This is because most of the photoacoustic techniques are based on a non-invasive measurement of the optical properties of the material under study. For example, photoacoustic imaging typically uses lasers in the near infrared to evaluate the strength and location of their absorption in biological tissue [5]. As the absorption coefficient of different tissue is also different, this method provides spectacular insights into the body. However, it is not applicable here, since the optical absorption coefficients of the materials in the visible and near-infrared range are determined by the coloration of the materials. 

Photoacoustic methods are only interesting if the generated sound signals depend on the chemical composition of the sample material, but less on its color. Fortunately, laser-induced material ablation, i.e., laser cutting itself, is known to generate material-specific sound waves [6]. Unlike other photoacoustic examination methods, this technique is invasive. A very short but powerful laser pulse generates “ablative piston signals”, which can be measured in the air above (i.e., noncontact) with ultrasonic microphones. This results in a very simple setup, which was first described by Leung and Tam in 1992 [6]. 

The potential of this technology for on-line process control or material discrimination was then recognized [7] and further investigated [8,9,10] by a research group at an ophthalmology clinic in Germany. They showed in [9] a successful material discrimination for biological tissue but also for three sorts of plastic (including PVC) by using cluster analysis in the frequency domain. In a more recent work, a discrimination accuracy between two sorts of plastic of 98 % was achieved with the cosine similarity technique [10].

Other groups investigated laser-acoustic monitoring for on-line ablation control of ceramics [11,12], as well as for welding processes [13,14] and laser grooving [15]. In addition, the possibility of photoacoustic discrimination of different (dental) materials in laser drilling [16,17,18] and skin tissue in burn surgery [19] was shown. The pursued approach is to evaluate the emitted sound energy, which is sufficient to distinguish two to three different materials.

However, all of them worked with powerful solid-state (Nd or Er:YAG) or gas lasers. These can easily achieve more than three orders of magnitude more optical energy per pulse and six orders of magnitude more power than the laser diode of the investigated laser cutter. From the scientific literature, it remains unclear if distinguishable laser-acoustic ablation signals can be generated with low-power laser diodes at all. 

Accordingly, this work has four objectives:

1. To show for the first time that laser-ablative sound is also formed by means of a low-power laser diode;
2. To investigate the material specificity of these sound signals;
3. To demonstrate the identification of PVC in a matrix of extrinsically similar plastics by means of a neural network evaluating these sound signals; and
4. To investigate the capabilities of this approach under real-life conditions.

In doing so, the contribution is split into three different parts: a physical one, where the ablation process is investigated by fast framing recordings and measurements of laser energy and ablation mass; second, a signal processing part, where the laser-ablative sound signal is investigated for its material specificity and other influencing factors; and in a last part, a simple neural network is set up, which is trained to classify seven different polymers and thereby identifying the problematic PVC. The neural network is also tested under non-ideal real-life conditions, such as ambient noise during recording.

Finally, all results are discussed in terms of their applicability to the initial aim of this contribution: the identification of problematic PVC in a low-cost desktop laser cutter.

## 2. Materials and Methods

### 2.1. General

#### Laser Diode

A blue (448 nm) laser diode (NDB7K75, Nichia Corp., Anan, Japan) with an optical output power of 3.5 W is focused on a material sample placed below. The diameter of the focal spot is approximately 0.2 mm. The laser diode is taken from a commercial laser cutter (“dreamcut 2”, Mr Beam Lasers GmbH, Munich, Germany). The default laser pulse salvo consists of 300 consecutive laser pulses with a width of 110 µs. The periodic time is 128 µs (duty cycle 86 %, frequency 7.8 kHz).

### 2.2. Materials

Relevant test materials are readily available plastics that can potentially be confused with PVC. Since the laser cannot process transparent, white, or blue materials (blue materials reflect the blue laser beam), these plastics must therefore also be available colored. The color black is particularly suitable, since the highest laser power is absorbed. Furthermore, black-colored plastics are currently a problem for the established sorting processes, as the added carbon absorbs most of the near-infrared light [2]. One sample of each of the following types of black-colored plastics was therefore investigated:

- Polymethyl methacrylate (PMMA);
- Polypropylene (PP);
- Polystyrene (PS);
- Polyvinyl chloride (PVC);
- Polyurethane (PU);
- Acrylonitrile butadiene styrene (ABS);
- Polyethylene terephthalate (PET);
- Polytetrafluoroethylene (PTFE);
- Polyethylene (PE);
- Polyoxymethylene (POM).

For the neural network and the fast-framing recordings, a subset of seven of these plastic types was used: PMMA, PP, PS, PVC, PU, ABS, and PET.

### 2.3. Physical Investigations of the Laser-Induced Ablation

#### 2.3.1. Fast Framing

The ablation of seven selected plastic materials was recorded by a fast-framing camera (FASTCAM Nova S16 1100 K-M-32 GB, Photron Deutschland GmbH, Reutlingen, Germany) to achieve a better understanding of this process and, hence, the laser-acoustic signal generation. As the event is only a few mm in size, a far-field microscope (QM100, Questar Corp., New Hope, PA, USA) was mounted on the camera. The laser diode itself illuminated the ablation process, which occurred during consecutive laser pulses of 110 µs length (the same specifications used for later laser-acoustic measurements). A frame rate of 10,000 frames/second, as well as a varying shutter speed, was applied. 

#### 2.3.2. Amount of Ablated Material

The amount of ablated material during one laser-pulse salvo was measured by comparing the mass of a PVC sample before and after the ablation with a scale (RC210D, Sartorius AG, Göttingen, Germany). 

#### 2.3.3. Laser Pulse Energy

The fluence of each laser pulse was measured with a laser-energy meter (J-10 MB-LE, Coherent Inc., Santa Clara, CA, USA). 

### 2.4. Laser-Acoustic Measurements

#### 2.4.1. Setup

**Figure 1:**
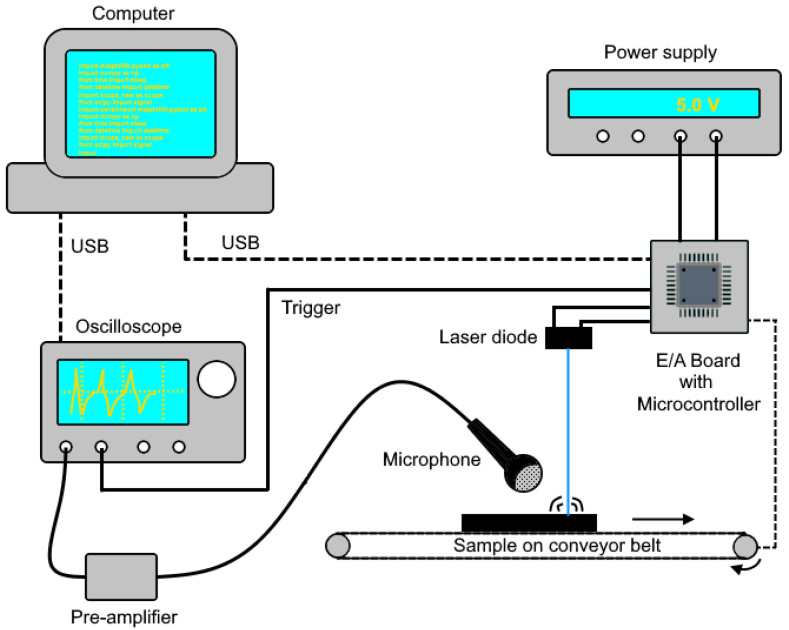
Experimental setup for laser-acoustic measurements.

The test setup for laser-acoustic measurements is schematically depicted in Figure 1. The material sample is on a conveyor belt, which allows several shots to be taken automatically. The voltage for the laser diode driver (5.0 V) is supplied by a DC power supply (IPS 2303S, RS components, Frankfurt am Main, Germany). A microcontroller (Pro Trinket 5 V, Adafruit Industries, New York City, NY, USA) switches a MOSFET (IRLZ44N, Infineon Technologies AG, Neubiberg, Germany) to power the laser diode, controls the conveyor belt, and releases a trigger signal to start the audio recording. 

The measurement microphone (MM310, Microtech Gefell GmbH, Gefell, Germany) is positioned at a 45° angle at the closest possible position from the focal spot (around 10 mm distance) at default. It is sensitive to frequencies up to 100 kHz. The audio signal is pre-amplified by a factor of 10 (8201 signal conditioner, YMC Piezotronics Inc., Yangzhou, China) before it is recorded by an oscilloscope (DPO2024B, Tektronix Inc., Beaverton, OR, USA).

The measuring process, including operating the microcontroller and reading the oscilloscope, is executed by a Python script. 

#### 2.4.2. Signal Processing: Data Acquisition and Preprocessing

**Figure 2:**
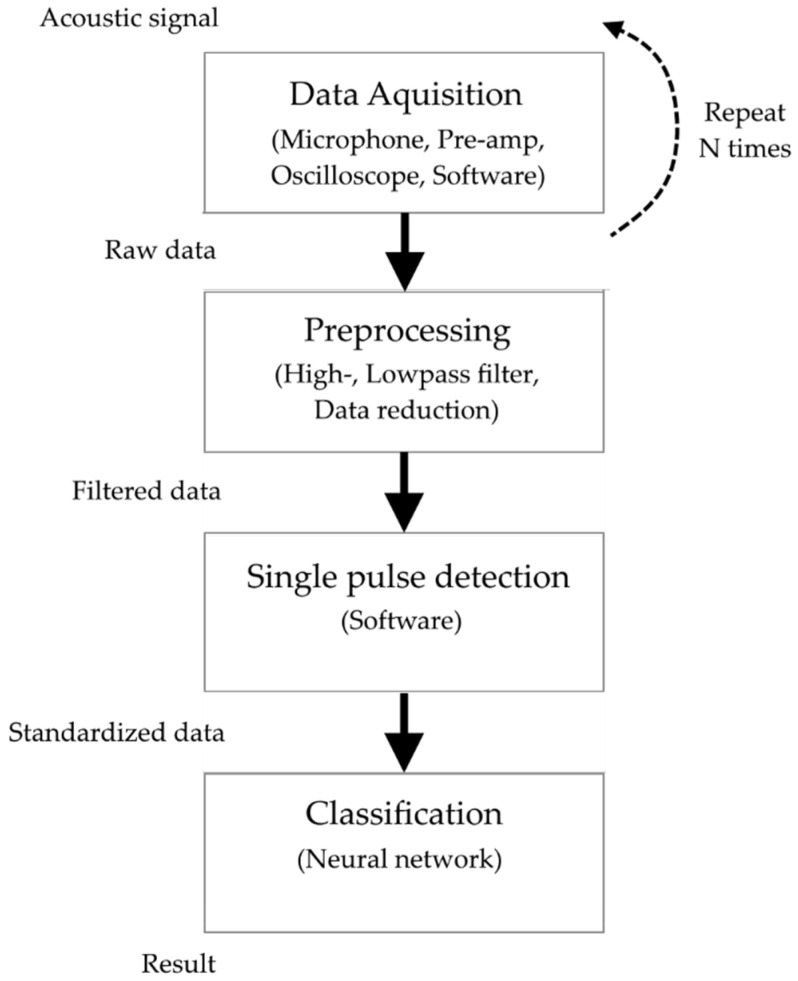
Flow chart of signal processing for the classification of the laser-acoustic material response.

**Figure 3:**
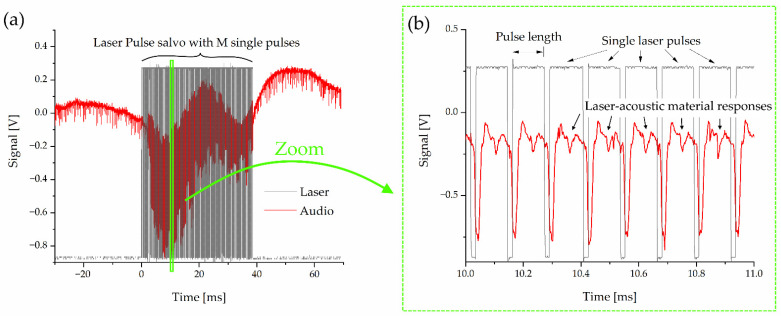
Signals recorded by the oscilloscope during one data-acquisition step (**a**): a laser-pulse salvo (**grey**) consisting of M (default: M = 300) single pulses lead to an acoustic response of the material (**red**). The zoom in (**b**) reveals single laser pulses (default length = 110 µs), each generating one high-frequency laser-acoustic material response.

The processing of the acoustic signal toward a standardized dataset fed into the neural network is schematically depicted in Figure 2. During one data-acquisition step, one laser-pulse salvo consisting of M single laser pulses generates the full laser-acoustic material response shown in Figure 3a. The magnified section in Figure 3b reveals repeating patterns of the audio signal. These are many high-frequency laser-acoustic material responses (LAMR), each of which can be assigned to a single laser pulse. These LAMR are evaluated and used to classify the material. To achieve this, further signal processing steps as shown in Figure 2 are necessary. 

Prior to this, the initial data-acquisition step can be repeated N times to increase the amount of gathered data, which is particularly important for neural network training requirements. Between each step, a conveyor belt shifts the material sample by a small distance (see Figure 1), which ensures that the sample surface conditions remain constant. N laser-pulse salvos, each containing M single laser pulses, therefore generate a maximum amount of up to:

p = M ∙ N,(1)

LAMR. The training (M = 300, N = 50) and test data (M = 300, N = 5) for each material sample hence contained a maximum of p_train_ = 15,000 and p_test_ = 1500 LAMR. As it becomes clear in the following section, the actual number of evaluable LAMR was much lower in some cases. 

One raw audio sample (see Figure 3a) is 100 ms long and contains 1.25 million data points (sampling rate 12.5 MHz). During the preprocessing step, electronic and ambient acoustic noise was removed by applying a low-pass (cutoff frequency 300 kHz) and a high-pass (cutoff frequency 1 kHz) Butterworth filter (2nd order), respectively. In doing so, the raw signal in Figure 3a loses the interfering low-frequency component, which makes the later detection more stable against noise. Thereafter, only every 10th data point was used without information loss to reduce the amount of data for further processing. 

#### 2.4.3. Signal Processing: Detection of LAMR

**Figure 4:**
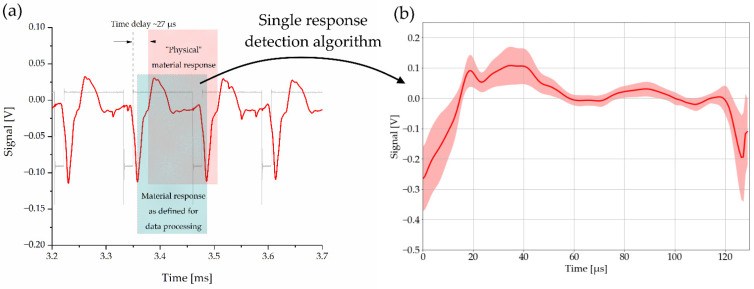
(**a**) Laser-acoustic signal during a laser salvo: the red rectangle marks a “physical material response”, which follows a single laser pulse after a time delay of ~27 µs (propagation time of the sound wave). The green box marks the single pulse response as defined for data processing. For a continuous pulse train, both contain the same data. Figure (**b**) shows the processed and averaged laser-acoustic material response of the “single pulse response-detection algorithm” applied on the signal in (**a**). The colored area around the graph corresponds to its 68% confidence band.

From the full audio signals, the LAMR were identified by their characteristic minima, whereas the region between two successive minima forms one LAMR with a typical length of 162 data points (see Figure 4a). Although the LAMR defined in this way do not correspond to the physical LAMR, they still contain the same data. The advantage of this method is that the individual pulses are independent of the propagation time of the sound signal, making the method less sensitive to the position of the microphone.

To exclude the initial heating phase and other interfering cases, minima that are too small in terms of magnitude (<10%) and pulses that are too short (<150 data points) or too long (>180) have been dropped during the preprocessing. The remaining pulses were then normalized to a length of 162. This means that longer pulses were cut and shorter ones were filled with zeros (zero padding). Figure 4b depicts the averaged results of this process from the audio signal in Figure 4a. 

A total of 66,011 LAMR were generated using the described procedure for the training data, ranging from only 3444 for PP up to almost the maximum of 15,000 for PET. These deviations can be explained by the fact that some materials generate much less of this LAMR or ones with too low amplitude. Between 5000 and 6000 LAMR were generated as test data for all materials depending on the varying recording conditions.

### 2.5. Classification

#### 2.5.1. Structure of the Neural Network

**Figure 5:**
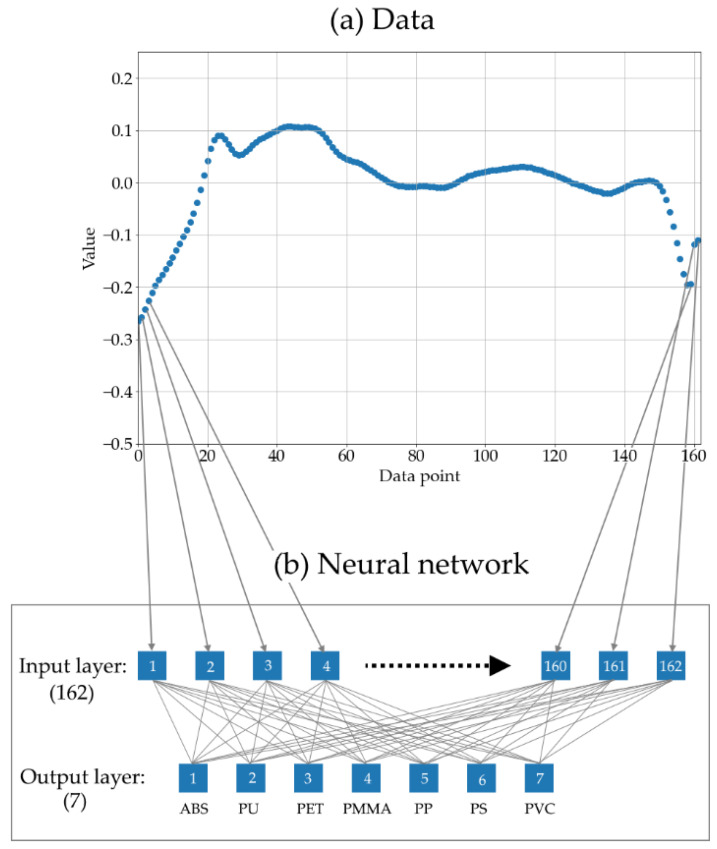
(**a**) One hundred and sixty-two float numbers representing a standardized LAMR are fed into the 162 nodes of the input layer of the neural network (**b**). The neural network is a single-layer feedforward network, where seven output nodes (each representing one type of plastic) are densely connected to all input nodes.

The present task is a multiclass classification with seven possible outcomes. For the evaluation of the data, the simplest possible neural network was programmed using the open-source software library “Keras”: a single-layer feedforward network with 162 input nodes and 7 output nodes, each corresponding to one type of plastic (Figure 5). A rectifier, “Adam”, and cross entropy were used as activation function, optimization algorithm, and definition for the loss function, respectively. The model was trained in 10 epochs using 10% of the training data for validation. 

A detailed analysis of the test results was performed using the confusion matrix. Accuracy and specificity were calculated for each species, accordingly.

#### 2.5.2. Pulse Salvo Classification

After each LAMR of one pulse salvo was classified by the neural network, the most frequently chosen type of plastic was assigned to the whole pulse salvo, thereby improving the prediction accuracy. 

#### 2.5.3. Stress Tests

The neural network and the pulse salvo classification were tested not only with data recorded under ideal conditions but also with purposefully perturbed data to check the stability of the classification. These include:

- Increased distance of the microphone (+5 mm);
- Changed angle of the microphone (60° instead of 45°);
- Red instead of black plastic (only for PU, PMMA, PP, PS, and PVC);
- Typical ambient noise during recording.

**Figure 6:**
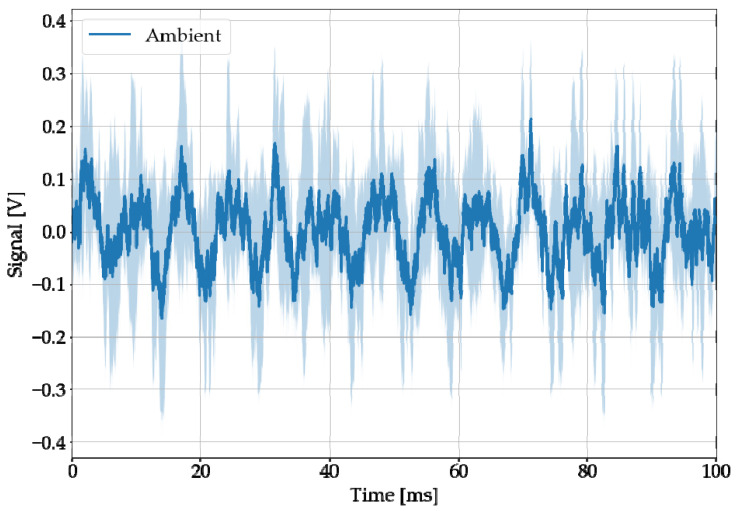
Averaged five audio recordings, when no laser but only loudspeakers playing typical office ambient noise were active.

Speakers playing the artificially created office sound “Office Ambience” (Copyright 2013 Iwan Gabovitch, CC-BY3 license) simulated the ambient noise. The audio file can be found in Audio S1. The loudspeakers were positioned and set up in such a way that, according to the subjective perception of the authors, a loud office background noise can be heard at the laser-acoustic test setup. The amplitudes of the sample recordings in Figure 6 are similar to these of LAMR.

#### 2.5.4. Confusion Matrices

The results of the neural network and pulse salvo classification were analyzed by confusion matrices, which contain the actual and the predicted types of plastic as rows and columns, respectively. From this data, the prediction accuracy (or sensitivity) of each type of plastic is calculated by the known formula:

Accuracy = True positives/Actual positives.(2)

Furthermore, the specificity for each type of plastic is calculated by the known formula:

Specificity = True negatives/Actual negatives.(3)

## 3. Results

### 3.1. Physical Background of Laser-Induced Ablation

**Figure 7:**
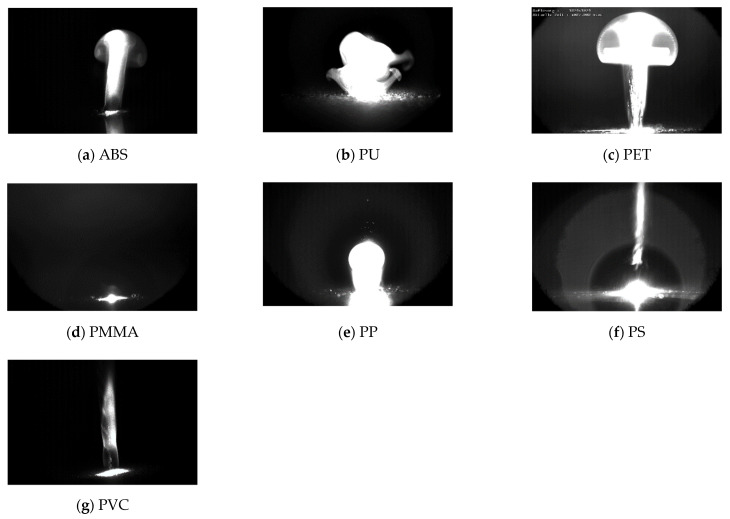
Photo of the ablation after the material received a pulse salvo containing 55 single laser pulses. Ablated particles reflect the laser light and make the plume visible. For scale: the size of the focal spot is between 0.2 and 0.5 mm in each case.

The fast-framing recordings in Figure 7 reveal the different ablation dynamics of seven different plastics, which originate from different quantity and speed of particle and gas emissions. Although the images only show the result of cumulative ablation of multiple pulses, it is plausible that individual laser pulses can also cause different ablation dynamics, too. Video S2 contains the recordings.

The ablated material from each pulse salvo was measured to be 6.8 µg on average in the case of black PVC. The fluence of each laser pulse was measured to be 116 ± 9 µJ or 232 ± 18 mJ/cm² referred to the focal spot. This corresponds to 34.8 mJ or 69.6 J/cm² for one pulse salvo. 

### 3.2. Analysis of the Laser-Acoustic Material Responses 

#### 3.2.1. General Description

**Figure 8:**
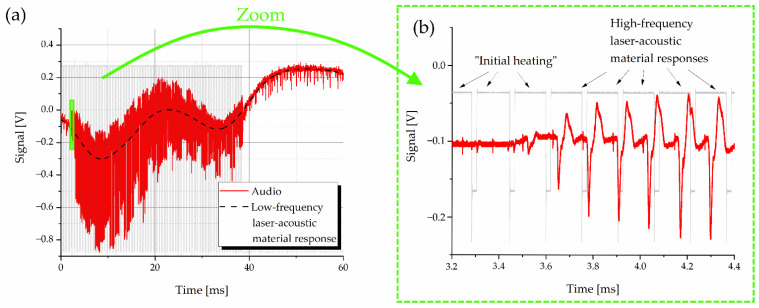
(**a**) Laser-acoustic material response recorded by the oscilloscope during a laser salvo on black PVC. It consists of a low-frequency component (**dashed black**) and a high frequency component, which corresponds to individual laser pulses and is more visible in the zoom (**b**). It can also be seen that the first laser pulses do not yet cause a high-frequency material response (“initial heating”).

The laser pulse salvo leads to an LAMR, which can be split in two different components: a low-frequency one (~50 Hz), which corresponds to the whole pulse salvo (Figure 8a), and many high-frequency ones (40–60 kHz) corresponding to single laser pulses (Figure 8b). 

Despite all materials showing the low-frequency LAMR, it was not taken into account in this contribution for two reasons. First, it is very susceptible to ambient noise compared to the high-frequency component. Second, one pulse salvo only generates one low-frequency LAMR compared to up to many high-frequency ones. The low-frequency component was filtered out during signal processing by a high-pass filter. All subsequent investigations were performed on the high-frequency LAMR, which will be only referred to as LAMR for short. 

Furthermore, the first laser pulses of each salvo were needed to just heat up the material without generating an evaluable sound signal (“initial heating” in Figure 8b). Therefore, a dense sequence of pulses is necessary to sum up their applied energies. The number of 300 pulses in each salvo was selected, as this always leads to predominantly stable material responses. 

#### 3.2.2. Pulse Length

**Figure 9:**
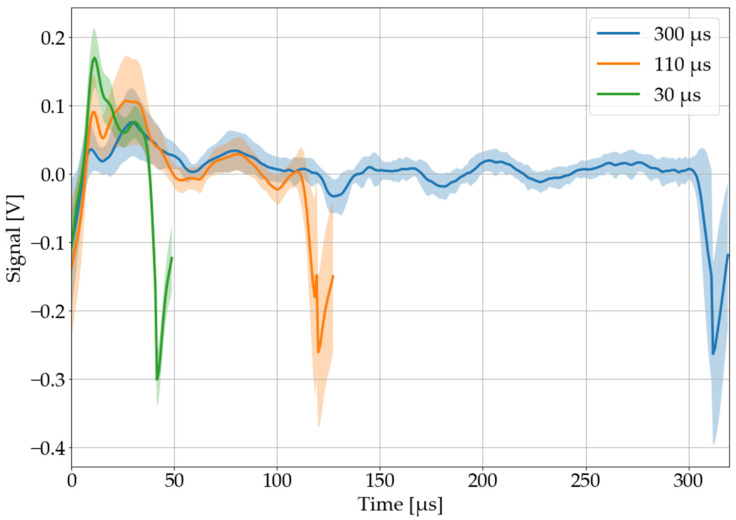
Recorded, processed, and averaged laser-acoustic material responses for laser pulses of different length. The sample is black polyvinyl chloride. The colored area around each graph corresponds to its 68% confidence band.

The pulse length is the most effective parameter to optimize the LAMR in terms of amplitude and information content. In Figure 9, the averaged LAMR of three different pulse lengths (30, 110, and 300 µs) are plotted. All pulse lengths were applied on black PVC in a periodic sequence with a pause between each pulse of 20 µs.

Longer pulses tend to have a lower amplitude, while shorter pulses may have less information content. For further investigations, the pulse length of 110 µs was selected for two reasons: first, there were only minor differences in amplitude between 30 and 110 µs, and furthermore, exactly this pulse length is already used in a commercial laser cutter (“dreamcut 2”, Mr Beam Lasers GmbH, Munich, Germany), which makes a later implementation easier. Further investigations with shorter pulse lengths may be the subject of future works.

#### 3.2.3. Sample Material

**Figure 10:**
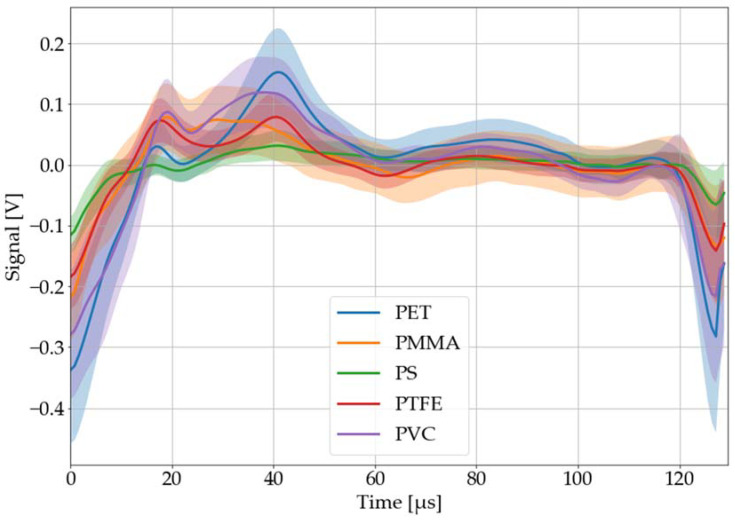
Black plastics with significant and potentially distinguishable laser-acoustic pulse responses: PET, PMMA, PS, PTFE, and PVC. The laser-acoustic responses were recorded, processed, and averaged. The colored area around each graph corresponds to its 68% confidence band.

**Figure 11:**
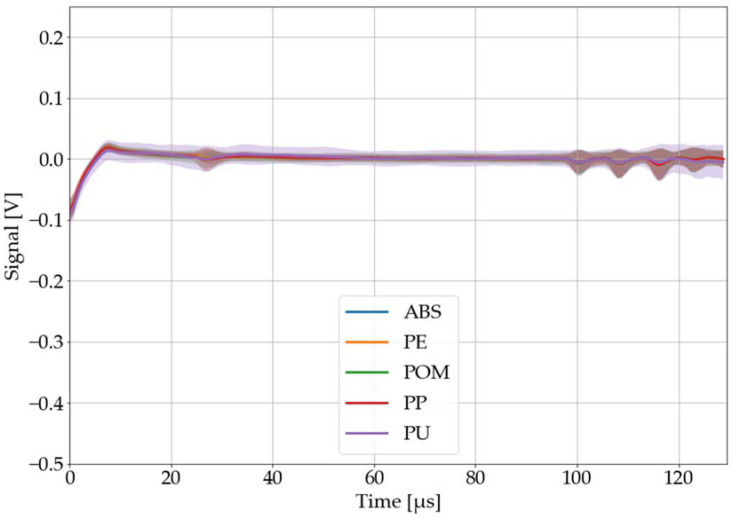
Black plastics with no and hardly distinguishable laser-acoustic material responses: ABS, PE, POM, PP, and PU. The laser-acoustic material responses were recorded, processed, and averaged. The colored area around each graph corresponds to its 68% confidence band.

The aim of the efforts is to distinguish the problematic material PVC from other plastics. Therefore, the recorded, processed, and averaged LAMR of all investigated black plastics are shown in Figure 10 and Figure 11. While Figure 10 depicts all materials with significant and potentially distinguishable LAMR (PET, PMMA, PS, PTFE, and PVC), Figure 11 displays all materials with little to no LAMR at all (ABS, PE, POM, PP, and PU). From these investigations, it is not clear why some materials have no LAMR at all. This should be subject for future work. 

For the identification of PVC by a neural network, a subset of seven plastic types was selected. Next to PVC, three representatives of each group were chosen: PET, PMMA, and PS with a distinct LAMR and ABS, PP, and PU with little to no LAMR. 

#### 3.2.4. Color of Sample Material

**Figure 12:**
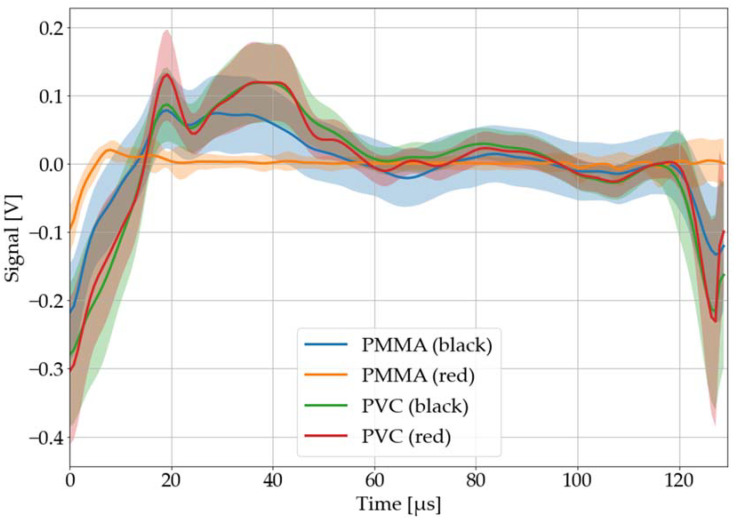
Recorded, processed, and averaged laser-acoustic responses for black and red PMMA and PVC. The colored area around each graph corresponds to its 68% confidence band.

The color of the sample material affects the absorption of the laser energy and, as it becomes clear in Figure 12, also LAMR to different amounts. While the LAMR of red PMMA becomes almost not existent, the one of PVC remains nearly the same. Therefore, this classification technique needs additional information about the color of the sample, which can be achieved by cheap color sensors. This contribution will initially focus on black material samples but will test whether red PVC is also recognized as such by the neural network for test purposes. 

#### 3.2.5. Number of Pulse Salvos

**Figure 13:**
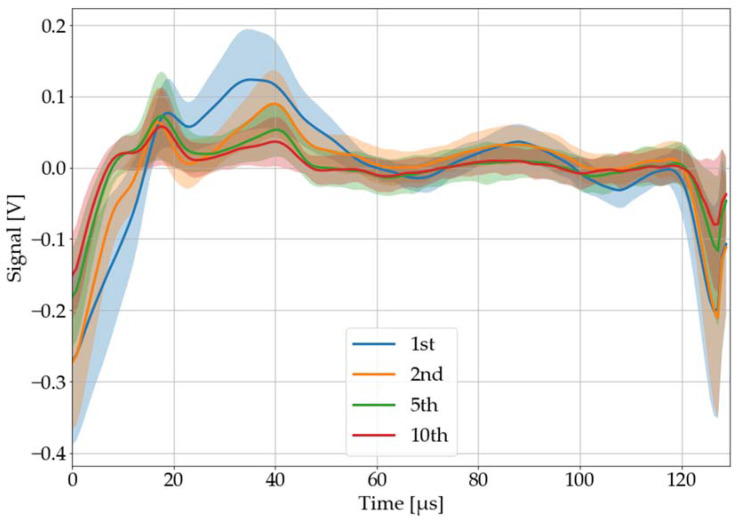
Recorded, processed, and averaged laser-acoustic material responses for black PVC, when successive laser-pulse salvos were shot at the same spot. The colored area around each graph corresponds to its 68% confidence band.

As can be seen in Figure 13, each successive laser-pulse salvo shot at the same spot decreases its generated LAMR a little bit. This can probably be attributed to the increasing depth of the ablation hole. To account for this, the material sample is shifted after each laser-pulse salvo automatically by a conveyor belt, so that surface conditions remain constant. 

#### 3.2.6. Position of the Microphone

**Figure 14:**
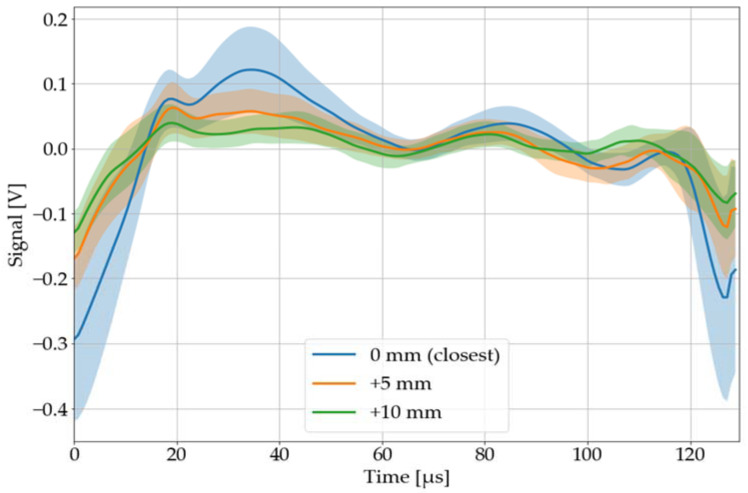
Recorded, processed, and averaged laser-acoustic material responses for black PVC, when the microphone positioned at an angle of 45° was moved away for 5 and 10 mm compared to its closest distance. The colored area around each graph corresponds to its 68% confidence band.

**Figure 15:**
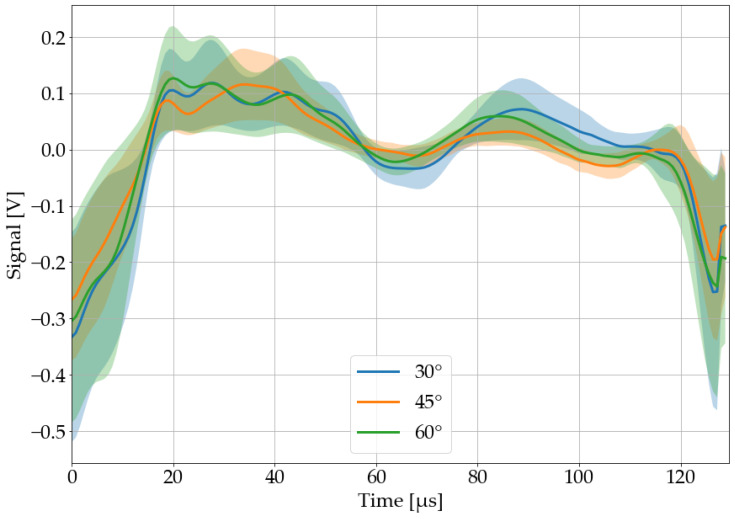
Recorded, processed, and averaged laser-acoustic material responses for black PVC, when the microphone was positioned at different angles. An angle of 0° would be parallel to the material surface, while 90° would be parallel to the laser beam. The colored area around each graph corresponds to its 68% confidence band.

The microphone is positioned at an angle of 45° and a distance of approximately 10 mm by default. Thereby, the distance of the microphone has a greater influence on the laser-acoustic material response than its angle, which can be seen in Figure 14 and Figure 15. The classification system will be stress tested with both variations. 

### 3.3. Performance of the Neural Network

#### 3.3.1. Single Pulse Classification

**Figure 16:**
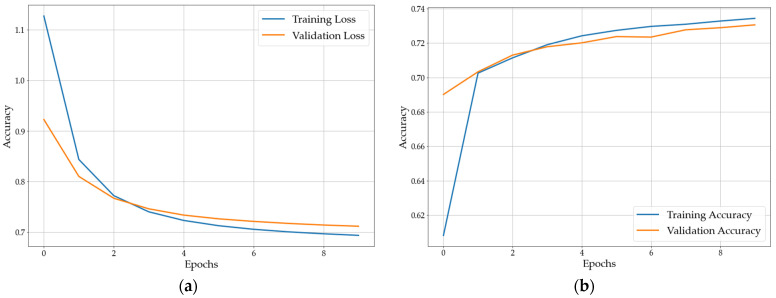
Training performance of the neural network: (**a**) loss function; (**b**) accuracy.

The very simple neural network described in Section 2.4.1 was trained as described with the standardized data in less than one minute on an office PC. The loss function and accuracy of the training and validation data converge after only 10 epochs (see Figure 16). This implies neither over- nor underfitting. The final loss and accuracy are 0.7 and 73%, respectively. The test data is classified 68% correctly.

A more detailed understanding of the classification performance enables the confusion matrix (see Table 1). As expected, there are major differences between the materials. While the plastics with a pronounced LAMR are correctly identified with an accuracy of over 50 and up to just under 90%, there are significantly lower values for all others (down to 5% in the case of PP). PVC can be identified with an accuracy of 78.2%. 

#### 3.3.2. Pulse Salvo Classification

The results can be further improved, when all pulses of one salvo are evaluated and the most frequently identified species were selected, accordingly. This leads to the following confusion matrix (Table 2). All materials with a distinct LAMR (PVC, PET, PMMA, and PS) are identified with perfect accuracy. For most of them, the classification is also perfectly specific. 

### 3.4. PVC Identification under Real-Life Conditions

The pulse salvo classification worked perfectly (100% accurate and specific) regarding the identification of PVC in a matrix of seven black plastics under ideal conditions. In the following, the model is tested with data recorded under less-than-ideal conditions. 

#### 3.4.1. Noise

The test accuracy (single pulse classification) dropped from 68 to 54%, when ambient noise was present during signal recording. The subsequent confusion matrix of the pulse salvo classification in Table 3 reveals that the identification of plastics with distinct LAMR mostly remain unaffected by ambient noise. Only one PVC salvo was incorrectly classified, thus its accuracy dropped to 80%. 

#### 3.4.2. Position of the Microphone

The test accuracy (single pulse classification) dropped from 68 to 36% when the distance of the microphone was increased by 5 mm. PVC was identified by pulse salvo classification with an accuracy of 100%, while its specificity dropped to 86%. Changing the microphone angle from 45 to 60°, on the other hand, leads to a decrease in test accuracy from 68 to 49%. The pulse salvo classification misinterpreted two salvos on PVC, thus its test accuracy dropped to 60% (specificity 100%). In conclusion, this method should be considered susceptible to changes in microphone position. 

#### 3.4.3. Color

Moreover, the model was tested (without training it on it) with red samples of PVC, PS, PP, PMMA, and PU. Although the single pulse test accuracy dropped to 40%, PVC was identified with 100% accuracy. Nevertheless, the system must be considered susceptible to the material color, since all other materials were almost completely misclassified (see Table 4).

## 4. Discussion

### 4.1. Material-Specific Laseracoustics with a Laser Diode

Laser-ablative sound waves are generally created by the explosive eruption of gas and particles. Fast-framing recordings have revealed that a pulsed laser diode can induce this kind of ablation behavior, despite having orders of magnitude less power than other laser sources.

Furthermore, the results have revealed for the first time that also material-specific, laser-ablative acoustic signals can be generated with a low-power laser diode. In contrast to previous work [7,8,9,10,11,12,16,17,18,19], however, this is only possible to a limited extent: the laser radiation must be very strongly focused to achieve the required area-related fluence. The individual pulses are also longer than in previous works to introduce sufficient energy. In addition, several pulses are necessary to first “preheat” the material. Gao et al. [20] also successfully applied a similar technique for photoacoustic imaging with a laser diode. For the following investigations, a laser-pulse salvo of 300 consecutive pulses, each 110 µs long, seemed sufficient and was chosen also for application-oriented purposes. 

These parameters led to the generation of two types of LAMR: a low frequency one, which is present at all materials, and a high-frequency one, occurring only when PET, PMMA, PVC, PS, and PTFE are processed. To the authors’ knowledge, this distinction has not yet been made in the literature. However, this contribution investigated only the high-frequency LAMR in depth, as it is more robust against noise, allows easier data acquisition, and occurs when the problematic PVC is processed. 

From this work, it remains unclear why exactly these materials show that kind of response but not the others (PE, PP, PU, ABS, and POM). Probably, the laser energy does not seem to be sufficient to ablate the material explosively in this case. Perhaps shorter laser pulses can also excite these materials to high-frequency LAMR. This should be the subject of future work. 

### 4.2. PVC Identification by a Neural Network Classification

#### 4.2.1. Strengths

This PVC identification system was developed for implementation in relatively cheap desktop laser cutters, where it is to protect the machine and the user from processing PVC. This system should therefore be markedly cost-effective. On the other hand, an examination time of up to one minute is tolerable. Considering these and other real-life operating conditions, the system was able to demonstrate the following strengths.

In a matrix of seven commercially available and extrinsically similar plastics, PVC was accurately identified by 78.2% under ideal conditions. The pulse salvo classification, where all LAMR in one laser pulse salvo are evaluated, even improved this result to a perfect 100%. A typical office ambient noise only reduced this prediction accuracy to 80%. This is due to the high-pass filter, which filters out most of the background noise, maintaining the ultrasonic LAMR. These results are comparable to existing work, where two organic polymers (polyamide and polyethylene) could be classified with an accuracy of 98% [10]. However, a much more powerful excimer laser (193 nm) and 4000 laser pulses (instead of 300 in the present case) were used there.

The evaluation of a test sample takes less than a minute, which is acceptable in this case. During one laser-pulse salvo (duration ~40 ms), 6.8 µg of material sample are ablated leading to concentrations in the surrounding air in the ppb range. This seems tolerable, considering that without the detection, the material would be processed for several minutes, releasing tens of thousands of times more potentially hazardous emissions.

#### 4.2.2. Limits and Areas for Improvement

The investigated technique shows some fundamental limits, and, in some aspects, it can still be improved. Improvements should be related to the recognition of both PVC and other plastics. Some materials show no (high-frequency) LAMR at all, which is why they cannot be identified reliably. The same mostly applies to other colors than black. However, it still has to be clarified whether these materials can then still be processed in the laser cutter at all and, if so, why they still do not generate LAMR.

The method seems susceptible to changes in microphone position. However, a fixed microphone with defined distance can certainly handle this problem.

The present test matrix contains only one physical sample of each plastic. Accordingly, the application on many physical samples with varying conditions and different content of dye and additives still needs to be proven.

The system may be trained or calibrated from time to time. A sample material with known LAMR could be used for this purpose. 

These results were still achieved with high-tech sensors. To develop a low-cost solution, the oscilloscope could be replaced by a simple A/D converter and the office PC by an embedded system. Piezo crystals may be useful as low-cost microphones in the ultrasonic range with high sensitivity.

Neural networks work very well in recognizing patterns. Accordingly, the result achieved is as expected, despite the simplest possible construction of the network. This can be attributed to the fact that the recorded audio data were preprocessed thoroughly. The decisive factor is that the individual pulses are determined by recognizing minima and are normalized to a predetermined length, which is computationally expensive, at least for embedded systems. Neural networks with more complex structures would probably be able to recognize material-specific patterns also in the full and untreated audio or frequency-shifted signal. This would enable a more general approach of identifying PVC in a greater test matrix containing also materials that do not even emit audio signals. The development of such a more powerful neural network will be the subject of future efforts.

#### 4.2.3. Impact

The investigated approach demonstrates that material classification is possible with very cost-effective laser diodes, which in turn results in more fields of application. However, the process certainly only has niche applications: first and foremost, the detection of PVC in desktop laser cutters. Time is not an issue here and an ablative laser diode is available anyway. On the other hand, the method is less useful for industrial plastic recycling or nondestructive material testing because it is destructive, too slow, and too susceptible.

In addition, applications in process monitoring are not possible yet, as the presented classification system is not real-time capable yet.

The technology can possibly also be used as a supplement to other methods. For example, the laser-acoustic approach could be combined with a classical camera and machine image recognition to enhance the number of materials that can be identified (especially different kinds of wooden materials, which play an important role in laser cutting and can require different processing parameters).

## 5. Conclusions

This contribution presents a method to classify black plastics using a laser-acoustic approach, a low-cost laser diode, and a very simple neural network. The discrimination of different plastic types is possible because of the different ablation characteristics of the plastics, which generate different acoustic footprints that are distinguishable by a neural network. The limitations and possibilities of this technology were subsequently discussed. An important feature of the presented classification system is the capability to reliably distinguish PVC from other, similar-looking materials. PVC can be detected by the neural network even when it is processed in a different color compared to the material with which the neural network was trained. Fast-framing images visualize the principle behind the presented method. Since PVC is commonly used for the manufacturing of mechanical parts, but as a source material of hazardous substances is not suitable for processing in laser cutters, the application of the laser-acoustic classification system can potentially improve the safety of desktop laser cutters.

Future research should investigate why some materials do not emit high-frequency LAMR yet and how the laser diode parameters may be tuned to achieve this. Furthermore, this system should be adapted to more cost-effective hardware, while the classification algorithm must be improved in terms of speed and computational effort. 

## Figures and Tables

**Table 1:** Confusion matrix of the test data with calculated accuracy (sensitivity) and specificity of each material.

True Values		Guesses							
	Total	ABS	PU	PET	PMMA	PP	PS	PVC	Accuracy
ABS	245	113	17	0	3	6	106	0	46.1%
PU	376	85	68	1	60	8	140	14	18.1%
PET	1530	50	10	1355	7	1	71	36	88.6%
PMMA	1463	77	24	1	1028	3	64	266	70.3%
PP	333	127	43	0	0	17	146	0	5.1%
PS	366	97	24	0	25	8	201	11	54.9%
PVC	1548	72	17	49	142	4	54	1210	78.2%
Specificity		91.7%	97.6%	98.8%	94.9%	99.4%	90.4%	93.0%	

**Table 2:** Confusion matrix of the test data with calculated accuracy (sensitivity) and specificity of each material, when pulse salvo classification is used.

True Values		Guesses							
	Total	ABS	PU	PET	PMMA	PP	PS	PVC	Accuracy
ABS	5	5	0	0	0	0	0	0	100%
PU	5	1	0	0	0	0	4	0	0%
PET	5	0	0	5	0	0	0	0	100%
PMMA	5	0	0	0	5	0	0	0	100%
PP	5	1	0	0	0	0	4	0	0%
PS	5	0	0	0	0	0	5	0	100%
PVC	5	0	0	0	0	0	0	5	100%
Specificity		93.7%	100%	100%	100%	100%	78.9%	100%	

**Table 3:** Confusion matrix of the test data recorded with ambient noise with calculated accuracy (sensitivity) and specificity of each material, when pulse salvo classification is used.

True Values		Guesses							
	Total	ABS	PU	PET	PMMA	PP	PS	PVC	Accuracy
ABS	5	0	0	0	0	0	5	0	0%
PU	5	0	0	0	0	0	5	0	0%
PET	5	0	0	5	0	0	0	0	100%
PMMA	5	0	0	0	5	0	0	0	100%
PP	5	0	0	0	0	0	5	0	0%
PS	5	0	0	0	0	0	5	0	100%
PVC	5	0	0	0	0	0	1	4	80%
Specificity		100%	100%	100%	100%	100%	58.8%	100%	

**Table 4:** Confusion matrix of the test data recorded with red samples with calculated accuracy (sensitivity) and specificity of each material, when pulse salvo classification is used.

True Values		Guesses							
	Total	ABS	PU	PET	PMMA	PP	PS	PVC	Accuracy
Red PU	5	0	1	0	0	0	4	0	20%
Red PMMA	5	0	0	0	0	0	5	0	0%
Red PP	5	2	1	0	0	0	2	0	0%
Red PS	5	0	0	0	5	0	0	0	0%
Red PVC	5	0	0	0	0	0	0	5	100%
Specificity		92.6%	96.1%	100%	83.3%	100%	69.4%	100%	

## Data Availability

The data that support the findings of this study are available upon reasonable request from the authors.

## Supplementary Materials

The following supporting information can be downloaded at https://www.mdpi.com/article/10.3390/s22208035/s1: Audio S1: Office Ambience Copyright 2013 Iwan Gabovitch, CC-BY3 license; Video S2: Fast framing video showing the ablation of different materials caused by a laser pulse salvo of 55 individual laser pulses.
